# Driving Forces of Liquid–Liquid Phase Separation in Biological Systems

**DOI:** 10.3390/biom9090473

**Published:** 2019-09-10

**Authors:** Boris Y. Zaslavsky, Luisa A. Ferreira, Vladimir N. Uversky

**Affiliations:** 1 Cleveland Diagnostics, Cleveland, OH 44114, USA; Luisa.Ferreira@Cleveland-Diagnostics.com; 2 Department of Molecular Medicine and Byrd Alzheimer’s Research Institute, Morsani College of Medicine, University of South Florida, Tampa, FL 33612, USA; 3 Laboratory of New methods in Biology, Institute for Biological Instrumentation, Russian Academy of Sciences, 142290 Pushchino, Moscow Region, Russia

**Keywords:** aqueous two-phase system, interfacial tension, liquid–liquid phase separation, phase-forming polymer, solvent features of water

## Abstract

Analysis of liquid–liquid phase separation in biological systems shows that this process is similar to the phase separation observed in aqueous two-phase systems formed by nonionic polymers, proteins, and polysaccharides. The emergence of interfacial tension is a necessary condition of phase separation. The situation in this regard is similar to that of phase separation in mixtures of partially miscible solvents. It is suggested that the evaluation of the effects of biological macromolecules on the solvent properties of aqueous media and the measurement of the interfacial tension as a function of these solvent properties may be more productive for gaining insights into the mechanism of liquid–liquid phase separation than the study of structural details of proteins and RNAs engaged in the process.

The liquid–liquid phase separation (LLPS) observed in the cytoplasm, nucleoplasm, mitochondrial matrix, and stroma of chloroplasts [1,2,3,4,5,6,7] is currently the research focus of multiple groups. The role of LLPS in the organization of cell biochemistry and its possible role [8] in fast cellular responses to external stimuli have attracted many researchers from different fields. The publications in the field are numerous and exponentially growing (see Figure 1). The majority of these publications are focused on the analysis of macromolecules (proteins and RNA/DNA) enriched in membrane-less organelles and their structural features [7,9,10,11,12,13,14,15,16,17], as well as on the analysis of model systems capable to isolate protein-rich phases under certain conditions in vitro as well as in vivo. Typically, the model systems used in such studies include different phase-separating proteins, such as tau-protein [18], elastin-like polypeptides [19], fused in sarcoma (FUS) [20], transactive response element (TAR) DNA-binding protein of 43 kDa (TDP-43) [21], structural γ-crystallins [22], polyQ-protein Whi3 [23], proteasomal shuttle factor UBQLN2 [24], nucleophosmin (NPM1) [25], Numb/Pon complex from Drosophila neuroblast [26], C9orf72 dipeptide repeat proteins [27], multivalent signaling proteins [28], and many others. There are several reports [29,30] indicating that the solvent properties of membrane-less organelles and protein-rich phases are different from those of the environment from which these phases originate.

It is well known that different nonionic polymers, proteins, and polysaccharides in their aqueous mixtures may form systems with two or more phases [31,32,33,34,35]. It is also known that the LLPS occurring in such mixtures when the concentrations of phase-forming polymers exceed certain thresholds typically leads to the phase-forming macromolecules being highly concentrated in different phases. If we ignore the solvent, aqueous in both phases, from the physicochemical point of view, the LLPS in such mixtures appears similar to that observed in mixtures of partially miscible solvents, such as water and butanol or methyl ethyl ketone. As an example, aliphatic alcohols such as butanol or pentanol are quite miscible with water until a particular concentration, beyond which the two mutually saturated solvents separate into two phases. The saturation concentration of water in n-butanol is 20.3%wt, and the saturation concentration of butanol in water is 7.4%wt. Any further addition of either solvent leads to an increasing volume of the corresponding phase, while the properties of the phases remain the same.

The situation is different for aqueous mixtures of two phase-forming polymers (including proteins and polysaccharides). The concentrations of the polymers in the two phases continues to change above the threshold concentration of phase separation, resulting in the formation of two-phase systems with properties different from those at lower concentrations of the polymers. Hence, the mixture of the same two phase-forming polymers in water can form a large variety of two-phase systems with properties dependent on the polymers concentrations.

It is important to note that for liquid–liquid phase separation to occur in water, as well as in any solvent mixture, the necessary and sufficient condition is the emergence of interfacial tension. The interfacial tension between organic solvent and water biphasic systems is well known [36] to increase with increasing dissimilarity of the two solvents. However, in any of these organic solvent–water biphasic systems, the value of interfacial tension is not affected by the increase in the organic solvent concentration. On the other hand, in aqueous two-phase systems formed by two polymers, it is well established experimentally [37,38,39,40] that the interfacial tension increases with increasing concentrations of the polymers, i.e., with increasing difference between the polymer concentrations in the two phases.

According to the recently reported [41] model of phase separation in aqueous mixtures of two polymers, the phase diagram may be described in terms of the polymers’ effects on the solvent features of water. The different properties of the coexisting phases in aqueous two-phase systems are successfully described by different solvent properties of water in the phases [42,43,44]. It should be emphasized that the Flory–Huggins theory of the incompatibility of polymers in solution considers the solvent solely as a diluent of unfavorable contacts between polymers and cannot be used to explain the LLPS in aqueous media. This is unsurprising, as the theory was stated [45] as inapplicable to polar systems. Therefore, attempts to use this theory for explanation of LLPS in biological systems seem to be counterproductive.

All the above considerations allow us to hypothesize that the emergence of interfacial tension and resulting LLPS in biological systems may be the consequence of different effects of phase-forming biopolymers (proteins, RNAs) on the properties of water in the cytoplasm, nucleoplasm, mitochondrial matrix, or stroma of chloroplasts. We suggest that studies of interfacial tension might be more productive for gaining deeper insights into the molecular mechanism of LLPS in biology than studies of unarguably important structural details of the macromolecules participating in and/or driving such LLPS.

## Figures and Tables

**Figure 1:**
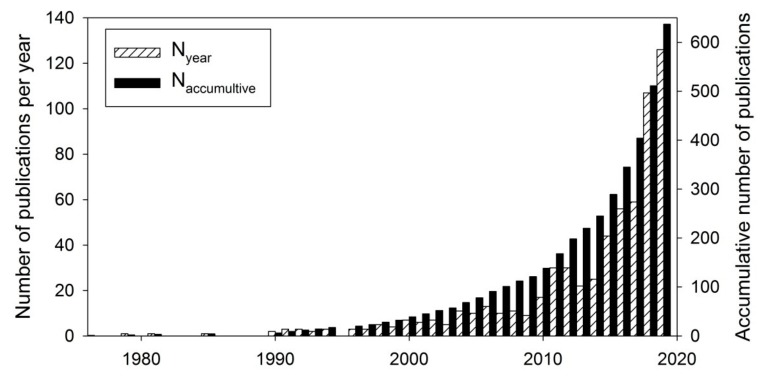
Increase in the number of publications dealing with liquid–liquid phase separation in protein solutions: Number of publications per annum (white crossed bars); accumulative number of publications (black bars). Data are based on the results of a PubMed search on August 19, 2019, using the search terms “liquid-liquid phase separation” and “protein”.
